# Supplementary material for: Predicting gene regulatory regions with a convolutional neural network for processing double-strand genome sequence information
Source: PLoS One. 2020 Jul 23;15(7):e0235748. doi: 10.1371/journal.pone.0235748 (PMC7377372; doi:10.1371/journal.pone.0235748)
Supplement: S1 Fig — (A) One-hot vectors that represent a DNA sequence. A 1-kbp DNA sequence is converted into a 1000×4 matrix. (B) A general convolutional network for sequence classifications. Only the first layer is shown, as an example. In the first layer, 9×4 kernels "scan" a sequence by striding base pair-by-base pair. Each "scan" is actually an element-wise multiplication of the values of a kernel (wmnhk) with values of 0 or 1 in a part of the sequence, yielding a scalar value represented by the black-to-white colored boxes shown below. By striding, scalar values are concatenated to a vector of size 992 (1000–9 + 1). Because the layer has multiple kernels (320 in this case), the output of the layer is 320 vectors. Maxpooling chooses maximum elements in each window. In this case, because the window size is 2, the total length of the sequence is halved by pooling. After fully connecting the layers, sigmoid operations yield output values between 0 and 1, which is compared with the class labels of "0" and "1". (PDF) [file pone.0235748.s001.pdf]

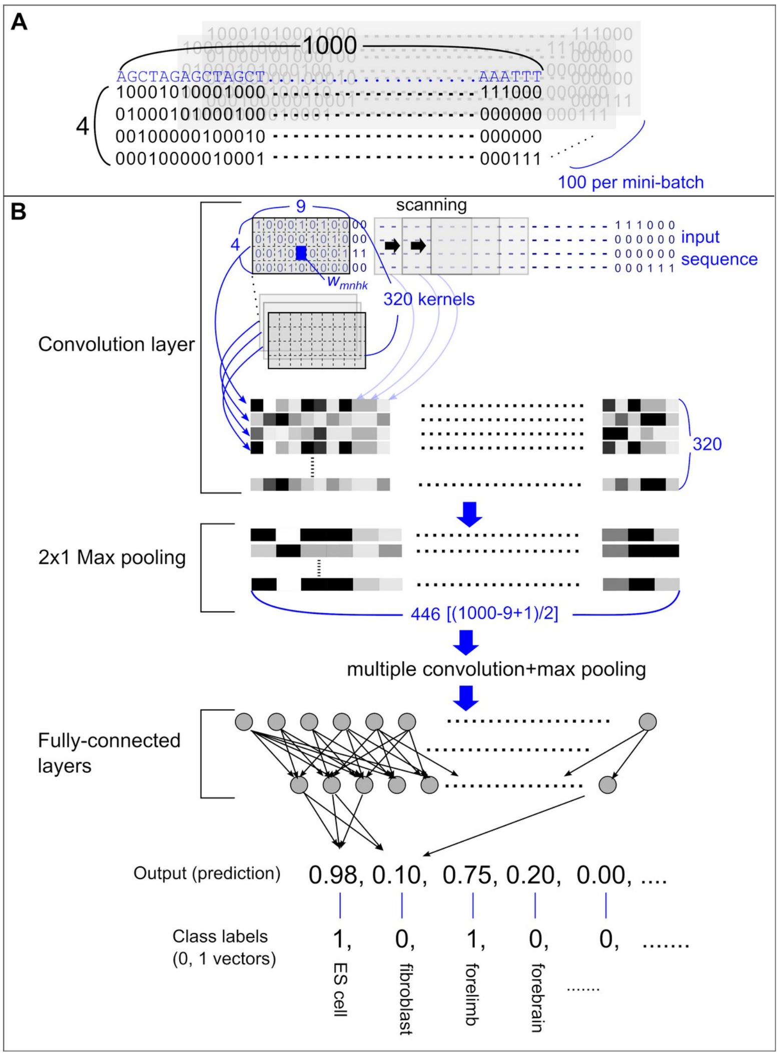

S1 Fig. Illustration of how a convolutional neural network reads DNA sequences. (**A**) One-hot vectors that represent a DNA sequence. A 1-kbp DNA sequence is converted into a 1000×4 matrix. (**B**) A general convolutional network for sequence classifications. Only the first layer is shown, as an example. In the first layer, 9×4 kernels "scan" a sequence by striding base pair-by-base pair. Each "scan" is actually an element-wise multiplication of the values of a kernel (w_{mnhk}) with values of 0 or 1 in a part of the sequence, yielding a scalar value represented by the black-to-white colored boxes shown below. By striding, scalar values are concatenated to a vector of size 992 (1000 – 9 + 1). Because the layer has multiple kernels (320 in this case), the output of the layer is 320 vectors. Maxpooling chooses maximum elements in each window. In this case, because the window size is 2, the total length of the sequence is halved by pooling. After fully connecting the layers, sigmoid operations yield output values between 0 and 1, which is compared with the class labels of "0" and "1".
