# Supplementary material for: Predicting gene regulatory regions with a convolutional neural network for processing double-strand genome sequence information
Source: PLoS One. 2020 Jul 23;15(7):e0235748. doi: 10.1371/journal.pone.0235748 (PMC7377372; doi:10.1371/journal.pone.0235748)
Supplement: S2 Fig — Boxplots of AUPRCs sorted by factors. The data were derived from nmeth.3547-S3.xlsx of Zhou & Troyanskaya (2015). Boxes, the lower-to-upper quantile values for the data; orange lines, the median; whiskers, the range of the data. Flier points, those past the end of the whiskers. Note that the prediction of CTCF binding sites (red rectangle) had the best performance among transcription factors. (PDF) [file pone.0235748.s002.pdf]

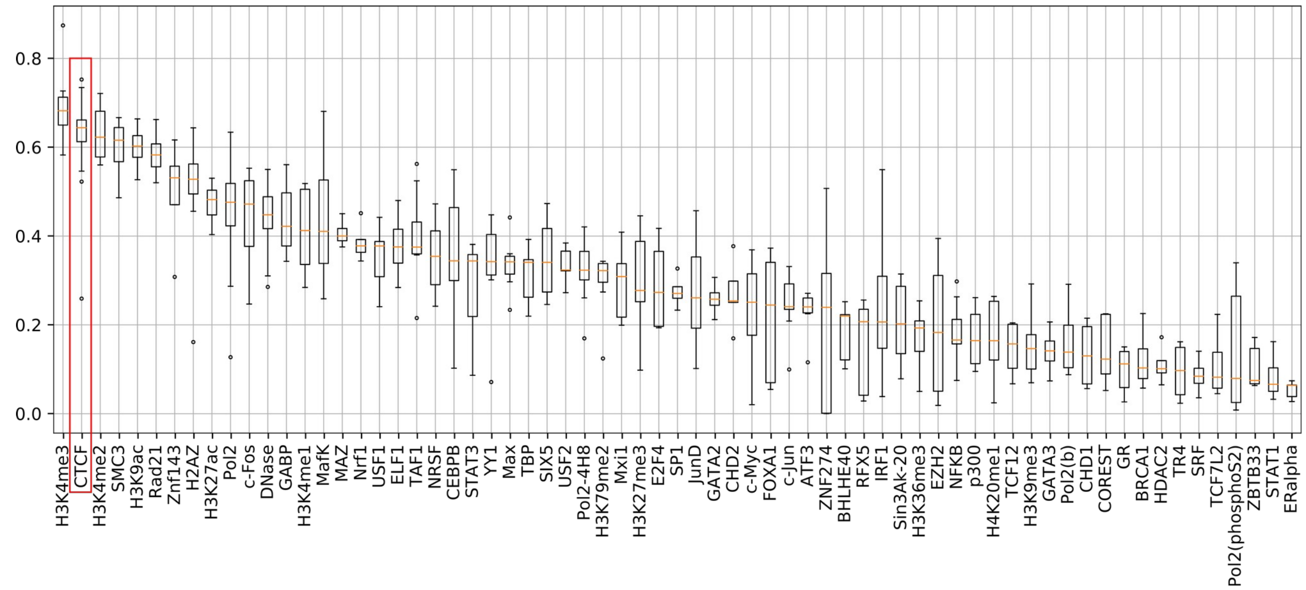


**S2 Fig. AUPRCs of the DeepSEA model.** Boxplots of AUPRCs sorted by factors. The data were derived from nmeth.3547-S3.xlsx of Zhou & Troyanskaya (2015). Boxes, the lower-to-upper quantile values for the data; orange lines, the median; whiskers, the range of the data. Flier points, those past the end of the whiskers. Note that the prediction of CTCF binding sites (red rectangle) had the best performance among transcription factors.
