# Supplementary material for: Predicting gene regulatory regions with a convolutional neural network for processing double-strand genome sequence information
Source: PLoS One. 2020 Jul 23;15(7):e0235748. doi: 10.1371/journal.pone.0235748 (PMC7377372; doi:10.1371/journal.pone.0235748)
Supplement: S3 Fig — AUPRCs are the prediction accuracy of conv4-FRSS trained with mm10 CTCF dataset. Black arrows indicate data with low AUPRCs but relatively high FRiP scores. The quality of these data is reasonably estimated with cFRiP. The validity of cFRiP is also supported by the better correlation with peak numbers. corr., Spearman’s correlation. (PDF) [file pone.0235748.s003.pdf]

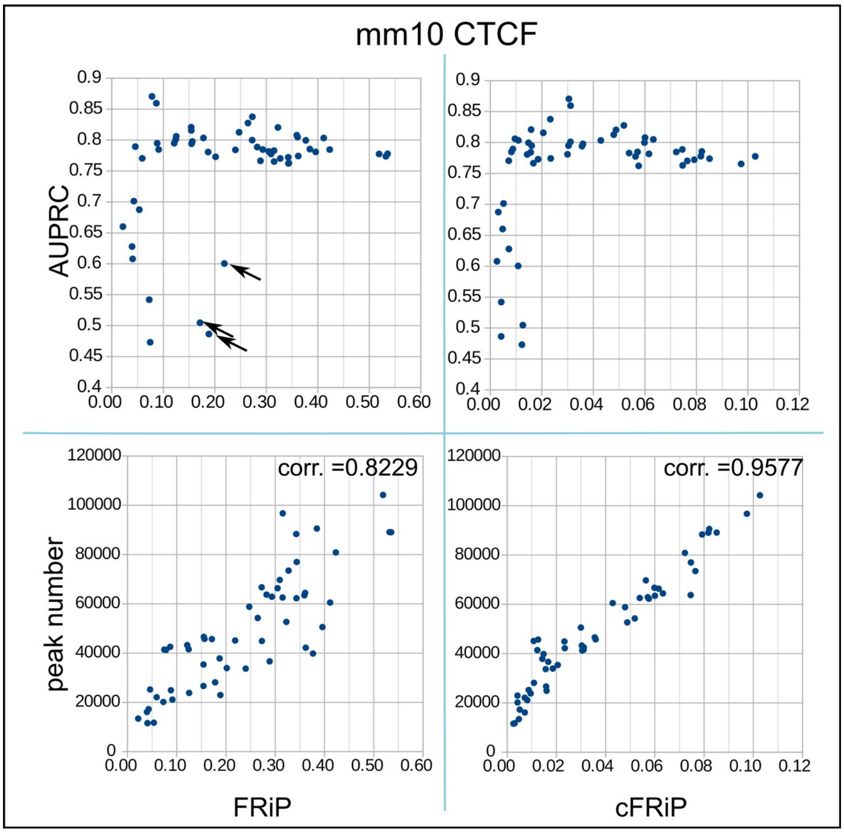

S3 Fig. Scatter plots for FRiP and corrected FRiP with AUPRCs (top panels) and peak numbers (bottom panels). AUPRCs are the prediction accuracy of conv4-FRSS trained with mm10 CTCF dataset. Black arrows indicate data with low AUPRCs but relatively high FRiP scores. The quality of these data is reasonably estimated with cFRiP. The validity of cFRiP is also supported by the better correlation with peak numbers. corr., Spearman’s correlation.
