# Supplementary material for: Predicting gene regulatory regions with a convolutional neural network for processing double-strand genome sequence information
Source: PLoS One. 2020 Jul 23;15(7):e0235748. doi: 10.1371/journal.pone.0235748 (PMC7377372; doi:10.1371/journal.pone.0235748)
Supplement: S4 Fig — (A, B) Plots of average AUPRCs for human and mouse DNase-seq data (A and B) and CTCF data (C and D) as a function of cutoff values of corrected FRiPs. Note that increase in cutoff values of the corrected FRiPs results in an increase of the average AUPRCs, at least to some extent, except as shown in c. Red line, cutoff values for the filtered data in Fig 2A and 2B. (PDF) [file pone.0235748.s004.pdf]

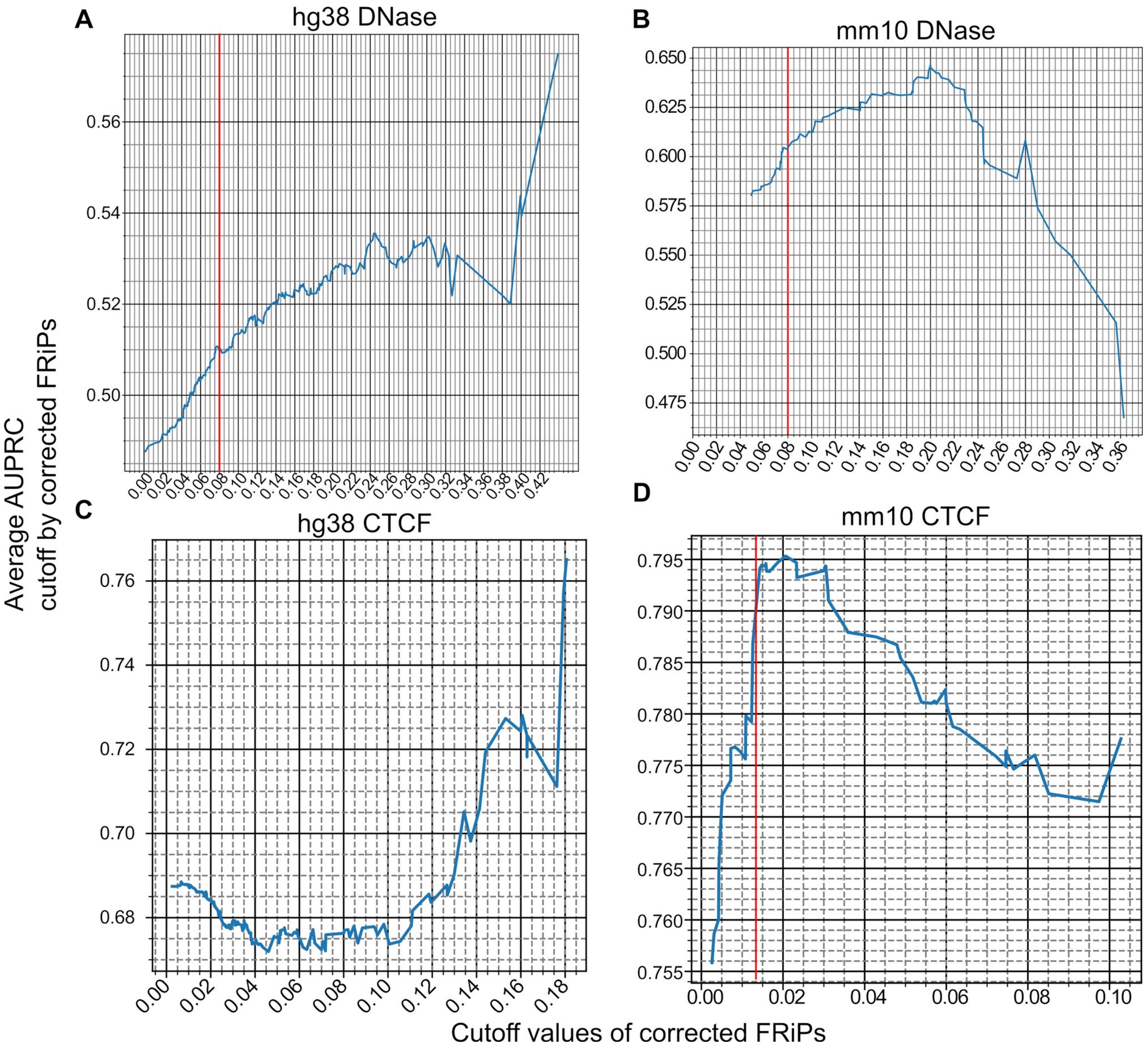

S4 Fig. The average of AUPRCs with various cutoff values of corrected FRiP. (**A, B**) Plots of average AUPRCs for human and mouse DNase-seq data (**A** and **B**) and CTCF data (**C** and **D**) as a function of cutoff values of corrected FRiPs. Note that increase in cutoff values of the corrected FRiPs results in an increase of the average AUPRCs, at least to some extent, except as shown in **c**. Red line, cutoff values for the filtered data in Fig. 2A and B.
