# Supplementary material for: Predicting gene regulatory regions with a convolutional neural network for processing double-strand genome sequence information
Source: PLoS One. 2020 Jul 23;15(7):e0235748. doi: 10.1371/journal.pone.0235748 (PMC7377372; doi:10.1371/journal.pone.0235748)
Supplement: S5 Fig — (A) CTCF binding distribution of mouse liver E14.5, as an example. Influential value, the values calculated by the class saliency extraction method; Prediction, CTCF binding sites predicted with conv4-FRSS; CTCF signal, CTCF ChIP-seq signals derived from ENCFF844ZSH; red rectangle, a region magnified in b. Note that the distribution of influential values is similar to that of the CTCF signal although the model is not directly trained with the signal data. (B) Comparison of influential values and CTCF binding motif. The CTCF binding motifs were detected with FIMO. Note that nucleotides with high influential value (orange) are aligned with the high information content sites in the CTCF motif (arrows). (PDF) [file pone.0235748.s005.pdf]

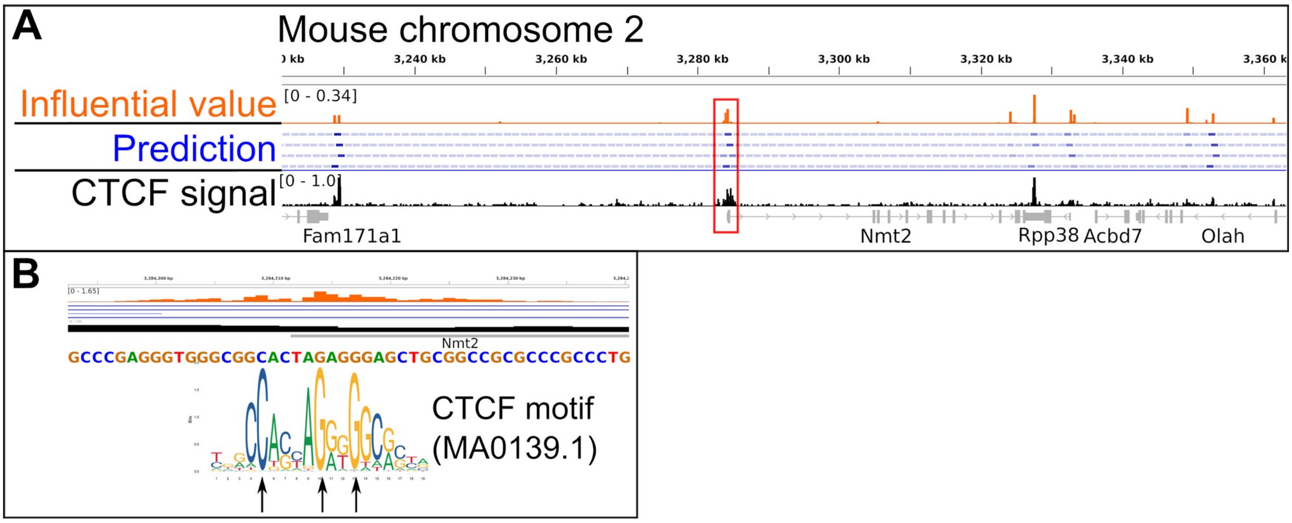

S5 Fig. Evaluation of informative sequences at the single-nucleotide level. (**A**) CTCF binding distribution of mouse liver E14.5, as an example. Influential value, the values calculated by the class saliency extraction method; Prediction, CTCF binding sites predicted with conv4-FRSS; CTCF signal, CTCF ChIP-seq signals derived from ENCFF844ZSH; red rectangle, a region magnified in b. Note that the distribution of influential values is similar to that of the CTCF signal although the model is not directly trained with the signal data. (**B**) Comparison of influential values and CTCF binding motif. The CTCF binding motifs were detected with FIMO. Note that nucleotides with high influential value (orange) are aligned with the high information content sites in the CTCF motif (arrows).
