# Supplementary material for: Predicting gene regulatory regions with a convolutional neural network for processing double-strand genome sequence information
Source: PLoS One. 2020 Jul 23;15(7):e0235748. doi: 10.1371/journal.pone.0235748 (PMC7377372; doi:10.1371/journal.pone.0235748)
Supplement: S6 Fig — Note that several kernels contain little information and thus appear empty. These kernels may suggest either that the kernel number was sufficiently set or that training was insufficient to optimize all kernels. (PDF) [file pone.0235748.s006.pdf]

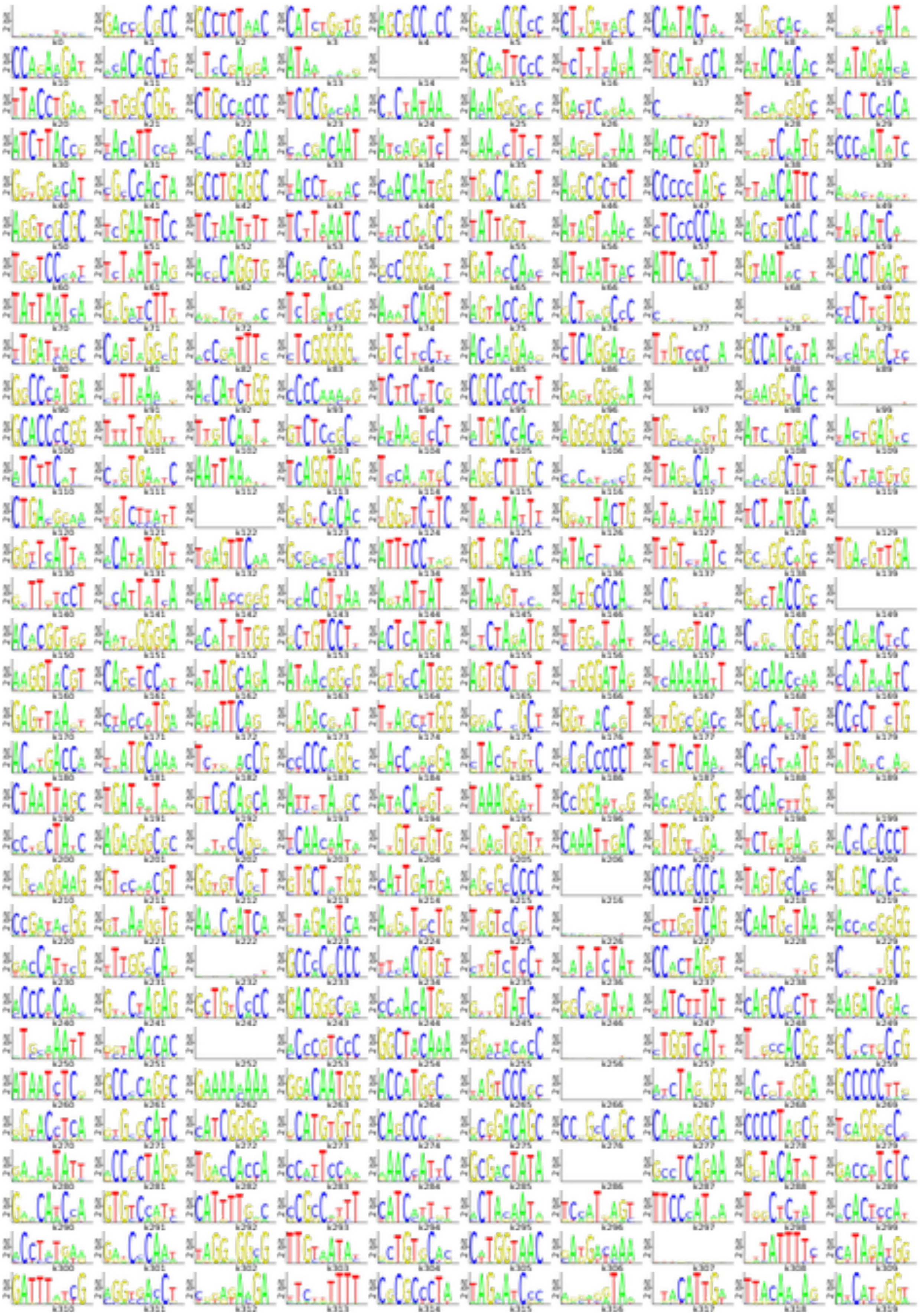

S6 Fig. All kernels of conv4-FRSS trained by a subset of the mouse DNase-seq data. Note that several kernels contain little information and thus appear empty. These kernels may suggest either that the kernel number was sufficiently set or that training was insufficient to optimize all kernels.
