# Supplementary material for: Predicting gene regulatory regions with a convolutional neural network for processing double-strand genome sequence information
Source: PLoS One. 2020 Jul 23;15(7):e0235748. doi: 10.1371/journal.pone.0235748 (PMC7377372; doi:10.1371/journal.pone.0235748)
Supplement: S1 Note — (PDF) [file pone.0235748.s007.pdf]

2. Supplementary Notes

Machine specification

OS: Ubuntu 16.04.4 LTS x86_64

GPU (×2):

```
  CUDA Driver Version / Runtime Version          9.0 / 8.0
  CUDA Capability Major/Minor version number:    6.1
  Total amount of global memory:                 12190 MBytes (12782075904 bytes)
  (28) Multiprocessors, (128) CUDA Cores/MP:     3584 CUDA Cores
  GPU Max Clock rate:                            1531 MHz (1.53 GHz)
  Memory Clock rate:                             5005 Mhz
  Memory Bus Width:                              384-bit
  L2 Cache Size:                                 3145728 bytes
  Maximum Texture Dimension Size (x,y,z)         1D=(131072), 2D=(131072, 65536), 3D=(16384, 16384, 16384)
  Maximum Layered 1D Texture Size, (num) layers  1D=(32768), 2048 layers
  Maximum Layered 2D Texture Size, (num) layers  2D=(32768, 32768), 2048 layers
  Total amount of constant memory:               65536 bytes
  Total amount of shared memory per block:       49152 bytes
  Total number of registers available per block: 65536
  Warp size:                                     32
  Maximum number of threads per multiprocessor:  2048
  Maximum number of threads per block:           1024
  Max dimension size of a thread block (x,y,z): (1024, 1024, 64)
  Max dimension size of a grid size    (x,y,z): (2147483647, 65535, 65535)
  Maximum memory pitch:                          2147483647 bytes
  Texture alignment:                             512 bytes
  Concurrent copy and kernel execution:          Yes with 2 copy engine(s)
  Run time limit on kernels:                     No
  Integrated GPU sharing Host Memory:            No
  Support host page-locked memory mapping:       Yes
  Alignment requirement for Surfaces:            Yes
  Device has ECC support:                        Disabled
  Device supports Unified Addressing (UVA):      Yes
  Device PCI Domain ID / Bus ID / location ID:   0 / 2 / 0
  Compute Mode:
     < Default (multiple host threads can use ::cudaSetDevice() with device simultaneously) >
```

CPU:

```
   Architecture:          x86_64
   CPU op-mode(s):        32-bit, 64-bit
```

```
Byte Order:            Little Endian
CPU(s):                20
On-line CPU(s) list:   0-19
Thread(s) per core:    1
Core(s) per socket:    10
Socket(s):             2
NUMA node(s):          2
Vendor ID:             GenuineIntel
CPU family:            6
Model:                 79
Model name:            Intel(R) Xeon(R) CPU E5-2640 v4 @ 2.40GHz
Stepping:              1
CPU MHz:               1200.093
CPU max MHz:           3400.0000
CPU min MHz:           1200.0000
BogoMIPS:              4801.79
Virtualization:        VT-x
L1d cache:             32K
L1i cache:             32K
L2 cache:              256K
L3 cache:              25600K
NUMA node0 CPU(s):     0-9
NUMA node1 CPU(s):     10-19
Flags:                 fpu vme de pse tsc msr pae mce cx8 apic sep mtrr pge mca cmov pat pse36
clflush dts acpi mmx fxsr sse sse2 ss ht tm pbe syscall nx pdpe1gb rdtscp lm constant_tsc
arch_perfmon pebs bts rep_good nopl xtopology nonstop_tsc aperfmperf eagerfpu pni pclmulqdq
dtes64 monitor ds_cpl vmx smx est tm2 ssse3 sdbg fma cx16 xtpr pdcm pcid dca sse4_1 sse4_2
x2apic movbe popcnt tsc_deadline_timer aes xsave avx f16c rdrand lahf_lm abm 3dnowprefetch
epb invpcid_single intel_pt kaiser tpr_shadow vnmi flexpriority ept vpid fsgsbase tsc_adjust
bmi1 hle avx2 smep bmi2 erms invpcid rtm cqm rdseed adx smap xsaveopt cqm_llc
cqm_occup_llc cqm_mbm_total cqm_mbm_local ibpb ibrs stibp dtherm ida arat pln pts
```

System memory: DDR4-2400 256GB
Data Storage: Crucial MX300SSD 2TB ×2 (RAID 0)
